# Dosimetric Comparison of Intraoperative Radiotherapy and SRS for Liver Metastases

**DOI:** 10.3389/fonc.2021.767468

**Published:** 2021-12-02

**Authors:** Davide Scafa, Thomas Muedder, Jasmin A. Holz, David Koch, Younéss Nour, Stephan Garbe, Maria A. Gonzalez-Carmona, Georg Feldmann, Tim O. Vilz, Mümtaz Köksal, Frank A. Giordano, Leonard Christopher Schmeel, Gustavo R. Sarria

**Affiliations:** ^1^ Department of Radiation Oncology, University Hospital Bonn, Bonn, Germany; ^2^ Department of Internal Medicine I, University Hospital Bonn, Bonn, Germany; ^3^ Department of Internal Medicine III, University Hospital Bonn, Bonn, Germany; ^4^ Department of Surgery, University Hospital Bonn, Bonn, Germany

**Keywords:** SRS, IORT, kilovoltage, liver metastases, intraoperative

## Abstract

**Purpose/Objectives:**

To perform a dosimetric comparison between kilovoltage intraoperative radiotherapy (IORT) and stereotactic radiosurgery (SRS) simulating both deep-inspiration breath-hold (DIBH) and free-breathing (FB) modalities for patients with liver metastases.

**Methods/Materials:**

Diagnostic computed tomographies (CT) of patients carrying one or two lesions <4 cm and who underwent surgery were retrospectively screened and randomly selected for the study. For DIBH-SRS, a gross target volume (GTV) plus planning target volume (PTV) were delineated. For FB-SRS, a GTV plus an internal target volume (ITV) and PTV were defined. Accounting for the maximal GTV diameters, a modified GTV (GTV-IORT) was expanded circumferentially to simulate a resection cavity. The best suitable round-applicator size was thereafter selected. All treatment plans were calculated homogeneously to deliver 40 Gy. Doses delivered to organs at risk (OAR) and target volumes were compared for IORT vs. both SRS modalities.

**Results:**

Eight patients encompassing 10 lesions were included in the study. The mean liver volume was 2,050.97 cm^3^ (SD, 650.82), and the mean GTV volume was 12.23 cm^3^ (SD, 12.62). As for target structures, GTV-IORT [19.44 cm^3^ (SD, 17.26)] were significantly smaller than both PTV DIBH-SRS [30.74 cm^3^ (SD, 24.64), p = 0.002] and PTV FB-SRS [75.82 cm^3^ (SD, 45.65), p = 0.002]. The median applicator size was 3 cm (1.5–4.5), and the mean IORT simulated delivery time was 45.45 min (SD, 19.88). All constraints were met in all modalities. Liver V_9.1_ showed significantly smaller volumes with IORT [63.39 cm^3^ (SD, 35.67)] when compared to DIBH-SRS [150.12 cm^3^ (SD, 81.43), p = 0.002] or FB-SRS [306.13 cm^3^ (SD, 128.75), p = 0.002]. No other statistical or dosimetrically relevant difference was observed for stomach, spinal cord, or biliary tract. Mean IORT D_90_ was 85.3% (SD, 6.05), whereas D_95_ for DIBH-SRS and FB-SRS were 99.03% (SD, 1.71; p = 0.042) and 98.04% (SD, 3.46; p = 0.036), respectively.

**Conclusion:**

Kilovoltage IORT bears the potential as novel add-on treatment for resectable liver metastases, significantly reducing healthy liver exposure to radiation in comparison to SRS. Prospective clinical evidence is required to confirm this hypothesis.

## Introduction

Liver metastases are a frequent cause of morbidity and mortality in oncology, with over 5% of all cancer patients developing these metastases at some point throughout the evolution of their disease and approximately 15% of them surviving 1 year after diagnosis (1). Therapeutics have substantially improved along the past few decades, while surgery still remains as the pivotal treatment for those patients with resectable disease (2).

Current surgical trends have migrated from larger and more aggressive resections to a rather tissue-sparing strategy, with comparable oncological outcomes and improved perioperative morbidity (3). In counterpart to the classical anatomy-based resections, where an entire segment would be excised, surgeons might find some difficulties when securing sufficient margin intending to preserve functional parenchyma (4). This circumstance may affect loco regional control and survival outcomes (5).

However, the optimal treatment strategy for these patients remains unclear. Despite that surgery in combination with modern chemotherapeutic agents confer adequate control and survival results (6), subtotal resections still pose an increased risk of local recurrence, thus higher mortality rates (7). Recent data indicate that radiotherapy (RT) with different modalities is accompanied by significant antineoplastic activity, either as a definitive or as a preoperative selective internal modality (8, 9).

In order to improve the postoperative prognosis of these patients, we propose intraoperative radiotherapy (IORT) as a novel adjuvant method worth exploring. According to our hypothesis, IORT would encompass in a single approach surgery and adjuvant radiotherapy, shortening treatment times while preserving the utmost healthy tissue. This dosimetric proof of concept investigates the differences between IORT and stereotactic radiosurgery (SRS) in a single fraction, in terms of feasibility and exposure of surrounding healthy tissue and organs at risk.

## Methods

### Patients and Procedures

Patients diagnosed with one or two liver metastases, who underwent non-anatomical surgery, were screened and randomly selected form the institutional database, excluding those with lesions larger than 4 cm. Preoperative diagnostic imaging sets (contrast CT) were retrieved. Radiotherapy target volumes were CT or MRI based, after rigid matching.

Regarding the treatment targets, gross tumor volume (GTV) was defined as any visible MRI T1-weighted metastatic lesion, and three different RT delivery situations were simulated, as follows. To simulate a deep inspiration breath-hold (DIBH-SRS), the GTV was expanded 5 mm homogeneously to create a planning target volume (PTV). For free-breathing SRS (FB-SRS), the GTV was expanded 10 mm craniocaudally and 5 mm radially to create an internal target volume (ITV), and thereafter, 5 mm was applied for the PTV. For IORT, a circumferentially modified GTV (GTV-IORT) was created accounting for the outermost GTV borders in order to simulate a resection cavity. This structure was adjusted lately to encompass the immediate larger suitable applicator. The healthy liver contour encompassed the entire organ minus GTV or GTV-IORT, respectively.

All treatment plans were prescribed according to a single 40-Gy scheme, emulating a previously published liver stereotactic radiosurgery (SRS) experience (8). As per institutional standards, for both SRS modalities, a homogeneous 99% PTV coverage should receive 99% of the intended dose (D_99%_ = 99%). A 90% volume coverage receiving 100% of the dose was defined for IORT plans (D_90%_ = 100%). Calculations were performed on Eclipse 13.6 for TrueBeam STx (Varian Medical Systems, Palo Alto, CA, USA). Intraoperative RT calculations were done on Radiance (GMV SA, Madrid, Spain), employing a Monte Carlo simulation for low-energy X-rays to be delivered with the IntraBeam600 intraoperative system (Carl Zeiss Meditec AG, Jena, Germany).

Constraints were adopted from a phase I dose-escalation study (10). The biliary tract tolerance was defined according to related publications (8, 11). Details on description per organ at risk (OAR) are compiled in **Table 1**.

**Table 1:** Organs at risk constraints.

Structure	D_max_	Volumetric Constraints
**Uninvolved liver (liver—GTV)**		700 cm^3^ <9.1 Gy
**Spinal cord**	14 Gy	<0.035 cm^3^ 10 Gy
		<1.2 cm^3^ 7 Gy
**Stomach**	12.4 Gy	<10 cm^3^ 11.2 Gy
**Biliary tract**	25 Gy	

Organs at risk and predefined tolerance doses according to Meyer et al. The D_max_ point was defined as 0.035 cm^3^.

GTV, gross tumor volume.

### Endpoints

Main endpoints include exposure differences between techniques for healthy liver (D_700cm3_ < 9.1Gy), surrounding organs at risk and target coverage.

### Statistical Analysis

Direct comparisons between mean values and standard deviation (SD) were performed for each variable. Median values and their corresponding ranges are given, unless otherwise stated. The statistical analysis of differences employed the Wilcoxon signed-rank test for continuous variables, assuming a significance level when p ≤ 0.05.

### Ethics Statement

This retrospective dosimetric study was dispensed from Institutional Review Board (IRB) approval due to its nature. All personal data were anonymized, and no related information is provided in this manuscript, in consonance with the principles of the Declaration of Helsinki.

## Results

### Patients and Planning Features

Eight patients encompassing 10 lesions were included in the study (**Table 2**). The mean liver volume was 2,050.97 cm^3^ (SD, 650.82), and mean GTV volume was 12.23 cm^3^ (SD, 12.62). As for target structures, mean GTV-IORT [19.44 cm^3^ (SD, 17.26)] was significantly smaller than both mean PTV DIBH-SRS [30.74 cm^3^ (SD, 24.64), p = 0.002] and mean PTV FB-SRS [75.82 cm^3^ (SD, 45.65), p = 0.002]. The median applicator diameter was 3 cm (1.5–4.5), and the mean IORT simulated delivery time was 45.45 min (SD, 19.88).

**Table 2:** Contouring structures and dosimetric features.

Structure		Mean	SD
**GTV (cm^3^)**		12.23	12.62
**GTV-IORT (cm^3^)**		19.44	17.26
**Liver (cm^3^)**		2050.97	650.82
**Liver-IORT (cm^3^)**		2031.68	645.05
**Liver-GTV (cm^3^)**		2038.65	648.22
**Dosimetric features**		**Mean/Median**	**SD/Range**
**PTV DIBH-SRS**	**Volume (cm^3^)**	30.74	24.64
	**D95% (Gy)**	39.61	0.68
	**D95% (%)**	99.03	1.71
**PTV FB-SRS**	**Volume (cm^3^)**	75.82	45.65
	**D95% (Gy)**	39.22	1.38
	**D95% (%)**	98.04	3.46
**GTV-IORT**	**D90% (Gy)**	34.12	2.42
	**D90% (%)**	85.30	6.05
**IORT-Irradiation time (min)**		45.47	19.88
**Applicator diameter (cm)**		3.00	1.5-4.5

Target and liver volumes expressed in cm^3^. Target coverage displayed in absolute Gy and relative (percental) values. The median applicator diameter and mean simulated irradiation time are additionally shown.

GTV, gross tumor volume; IORT, intraoperative radiotherapy; PTV, planning target volume; DIBH, deep inspiration breath hold; SRS, stereotactic radiosurgery; FB, free-breathing; SD, standard deviation.

**Figure 1:**
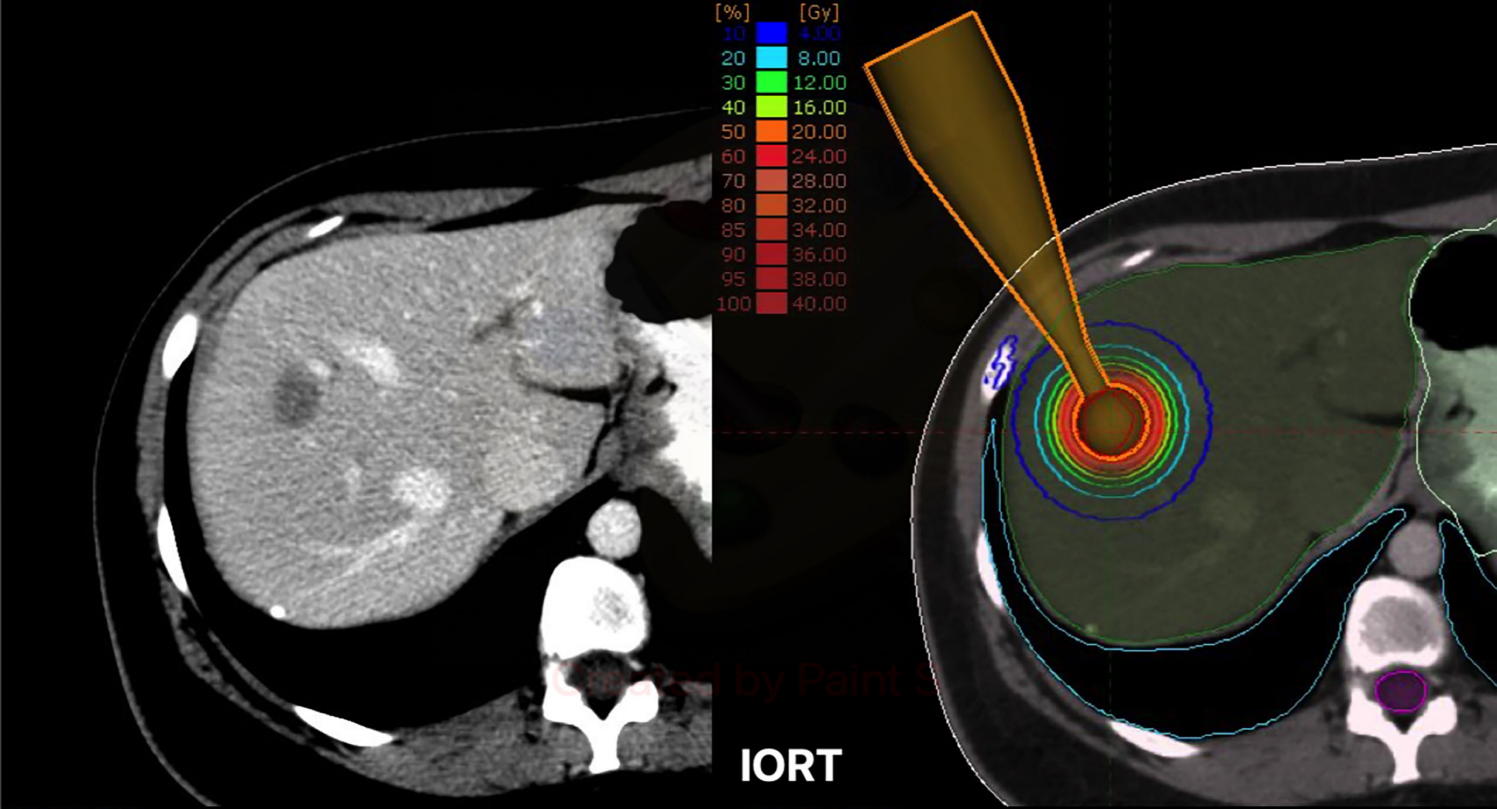
Left: Exemplary case with a single liver metastasis in segments VII-VIII. Right: Exemplary dose distribution profile with isodose lines for a simulated resection cavity. IORT, intraoperative Radiotherapy.

Liver V_9.1_ showed significantly lower exposure with IORT [63.39 cm^3^ (SD, 35.67)] when compared to DIBH-SRS [150.12 cm^3^ (SD, 81.43), p = 0.002] or FB-SRS [306.13 cm^3^ (SD, 128.75), p = 0.002; **Figure 1**]. For the remaining constraints, IORT was compared with DIBH-SRS and FB-SRS, and the results are detailed as follows. Respectively, mean results in Gy at spinal cord of 0.035 cm^3^ were 0.0, 0.0 (p = 0.371), and 0.16 (SD, 0.46, p = 0.371); spinal cord of 1.2 cm^3^, no significant exposure; stomach of 10 cm^3^, 0.0, 0.0 (p = 0.371), and 0.03 (SD, 0.08, p = 0.181); stomach D_max_, no accountable exposure; and biliary tract D_max_, 2.04 (SD, 3.25), 0.001 (SD, 0.004, p = 0.042), and 0.09 (SD, 0.29; p = 0.036; **Table 3**).

**Table 3:** Organs at risk exposure.

Constraints		Mean	SD
**Liver V9.1/700 cm^3^**	**IORT (cm^3^)**	63.39	35.67
	**DIBH-SRS (cm^3^)**	150.12	81.43
	**FB-SRS (cm^3^)**	306.13	128.75
**Spinal cord 0.035 cm^3^/7 Gy**	**IORT (Gy)**	0.00	0.00
	**DIBH-SRS (Gy)**	0.00	N.A.
	**FB-SRS (Gy)**	0.16	0.46
**Spinal cord 1.2 cm^3^/10 Gy**	**IORT (Gy)**	0.00	0.00
	**DIBH-SRS (Gy)**	0.00	N.A.
	**FB-SRS (Gy)**	0.00	0.00
**Stomach 10 cm^3^/11.2Gy**	**IORT (Gy)**	0.00	0.00
	**DIBH-SRS (Gy)**	0.00	N.A.
	**FB-SRS (Gy)**	0.03	0.08
**Stomach Dmax 12.4Gy**	**IORT (Gy)**	0.00	0.00
	**DIBH-SRS (Gy)**	0.00	0.00
	**FB-SRS (Gy)**	0.00	0.00
**Bilary tract 25Gy**	**IORT (Gy)**	2.04	3.25
	**DIBH-SRS (Gy)**	0.00	0.00
	**FB-SRS (Gy)**	0.10	0.29

Volumetric or maximum organs at risk exposure for all three explored modalities.

SD, standard deviation; IORT, intraoperative radiotherapy; DIBH, deep inspiration breath hold; FB, free breathing; SRS, stereotactic radiosurgery, NA, not assessable.

**Figure 2:**
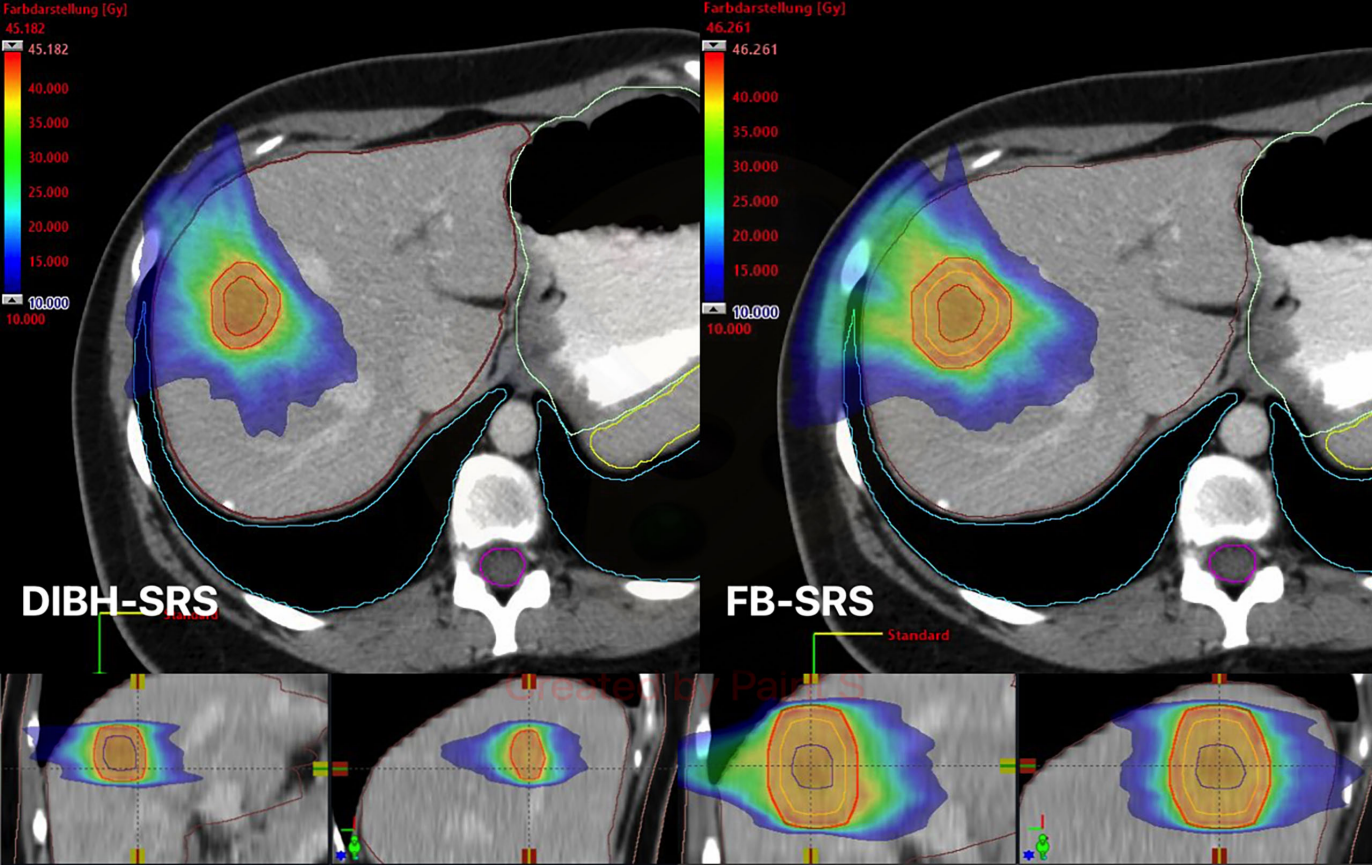
Exemplary case of dose distribution with color wash display for both DIBH-SRS (left) and FB-SRS (right). DIBH, deep inspiration breath hold; SRS, stereotactic radiosurgery; FB, free-breathing; GTV, gross tumor volume (blue line); ITV, internal target volume (orange line); PTV, planning target volume (red line).

Regarding target coverage, mean IORT D_90_ was 85.3% (SD, 6.05), whereas D_95_ for DIBH-SRS and FB-SRS were 99.03% (SD, 1.71; p = 0.042) and 98.04% (SD, 3.46) p = 0.036), respectively (**Figure 2**).

## Discussion

Strategies for improving clinical outcomes in patients carrying liver metastases have evolved over the past few decades. The standard management of these patients include surgery and systemic therapy. Surgery de-escalation through non-anatomical resection has recently been reported to be at least comparable to anatomical resections while sparing functional liver tissue (3). This is a relevant finding for patients undergoing several chemotherapy cycles along the course of their disease. In this sense, defining the role of radiotherapy results is compulsory, before including it as an adjuvant approach against this malignancy.

The utility of radiotherapy as stereotactic body radiotherapy (SBRT) has caused a resurge of the specialty in this scenario. Recent clinical data are progressively clarifying the differences between SBRT with other strategies, namely, trans-arterial chemoembolization (TACE), showing at least comparable outcomes in terms of toxicity and control outcomes (12). The rationale for selecting IORT and SRS as treatment schemes lies on the capacity of reproducing a surgical cavity from a tangible target structure. Furthermore, the recently reported 40-Gy single fraction eases a direct dosimetric evaluation of both modalities, with the added value of a clinically proven safe and effective reference (8).

Certainly, some considerations must be taken before deciding for a strategy above the other. In a set of patients who usually carry chronic liver damage, correctly assessing the patient’s baseline conditions is of great relevance before deciding for a therapeutic procedure. Radiation-induced liver disease (RILD) is one of the major pitfalls related to SBRT and hepatic malignancies (13). In this regard, reducing the exposed healthy liver tissue is of particular interest. Results from an earlier pilot trial assessing brachytherapy through an Ir^192^ high dose-rate (HDR) afterloader with interstitial catheters for non-resectable tumors demonstrated the feasibility and safety of this procedure. Afterwards, dosimetric analyses have further supported these findings, when comparing HDR to SBRT (14, 15).

Brachytherapy demonstrated to be a reliable option for treating patients in this setting. A retrospective study assessing the outcomes of 48 patients with hepatocellular carcinoma (HCC) treated with a single 15-Gy HDR fraction and posteriorly simulating an SBRT delivery reported a median PTV of 9.9 cm^3^ (SD, 12.9) and a V_10_ exposure of 92.8 cm^3^ (SD, 73.1) for the HDR cohort (16). An indirect comparison with our IORT results, accounting for larger target volumes (19.44 cm^3^), higher applied doses (40 Gy), and lower healthy liver exposure (V_9.1_ = 63.39 cm^3^), suggests that IORT would yield both superior delivery and tissue sparing in comparison to HDR. However, this method should be deemed as hypothesis generating, and a proper comparative dosimetric study should be performed before supporting any assumption.

The assessed OARs encompass the most critical to evaluate, according to the particular situation of the patients included in this work. This might be highly variable, depending on the tumor location and anatomical situation of other structures. Based on our findings, no intestinal segment or other surrounding organs were considered for reporting, as the doses delivered to these structures were negligible.

Regarding target volume coverage, a significant difference was observed in detriment of IORT. Respecting this, a major drawback related to the planning system was found: the underdosing area corresponds to the applicator’s neck, which does not show relevant dose absorption at the target volume. Furthermore, the IORT planning software does not allow modifying the target volume as a hollow structure, which would be optimal to improve the assessment of the plan. Nonetheless, these differences could be disregarded due to the real conditions that the practitioner might face during surgery. The area related to this underdosing represents the entry path of the applicator, which will be cleared of any vital tissue. It is most relevant to assure a full contact between the applicator and the surface to irradiate.

Limitations to this study encompass its retrospective nature and inherent features. Performing a dosimetric comparison between diagnostic GTV imaging and a simulated resection cavity might be subject of bias and not reproducible during a surgical procedure. However, this method allows a direct dosimetric comparison between both delivery techniques. Defining the actual surface to treat must be performed *in situ* under surgeon’s guidance. In addition, the relative biological effectiveness (RBE) factor was not included among these calculations in order to ease comparing both techniques. Noteworthy, RBE is not to be considered a rigid factor and might be subject to change according to different variables, including depth of delivery. Furthermore, it has not been clinically validated until now. Nevertheless, we recommend considering this variable during plan calculations, according to previous dosimetric and *in vitro* investigations (17). Taken together, this study provides relevant information for daily practice by providing dose-distribution profiles, which should be considered for clinical prospective evaluations.

## Conclusion

IORT could yield lower healthy liver tissue radiation exposure after non-anatomical liver metastasectomy, in comparison to SRS. Clinical prospective trials are warranted to confirm this hypothesis.

## Data Availability Statement

The original contributions presented in the study are included in the article/**Supplementary Material**. Further inquiries can be directed to the corresponding author.

## Ethics Statement

This retrospective dosimetric study was dispensed from Institutional Review Board (IRB) approval due to its nature. All personal data were anonymized, and no related information is provided in this manuscript, in consonance with the principles of the Declaration of Helsinki.

## Author Contributions

DS: study conceptualization and design, data production, collection and statistical analysis, manuscript drafting, and editing. TM: study design, data production, and analysis. JH: manuscript review and editing. DK: manuscript review and editing. YN: manuscript review and editing. SG: manuscript review and editing. MG-C: manuscript review and editing. GF: manuscript review and editing. TV: manuscript review and editing. MK: manuscript review and editing. FG: study design, manuscript review, and editing. LS: study conceptualization and design, manuscript review, and editing. GS: study conceptualization and design, data production, collection and statistical analysis, manuscript drafting, and editing. All authors contributed to the article and approved the submitted version.

## Funding

This study was supported by institutional funds.

## Conflict of Interest

DS has stocks from AstraZeneca GmbH. MG-C received travel expenses and honoraria from Bristol-Myers Squibb, Roche, MSD, IPSEN, Eisai, Amgen, and Servier not related to this work. FG received research grants and travel expenses from ELEKTA AB; grants, stocks, travel expenses, and honoraria from NOXXON Pharma AG; research grants, travel expenses and honoraria from Carl Zeiss Meditec AG; travel expenses and honoraria from Bristol-Myers Squibb, Roche Pharma AG, MSD Sharp and Dohme GmbH and AstraZeneca GmbH; non-financial support from Oncare GmbH and Opasca GmbH, not related to this work. LS GS received personal fees from Carl Zeiss Meditec AG, personal fees from Roche Pharma AG, personal fees from MedWave Clinical Research BV, and travels expenses from Guerbet GmbH.

The remaining authors declare that the research was conducted in the absence of any commercial or financial relationships that could be construed as a potential conflict of interest.

## Publisher’s Note

All claims expressed in this article are solely those of the authors and do not necessarily represent those of their affiliated organizations, or those of the publisher, the editors and the reviewers. Any product that may be evaluated in this article, or claim that may be made by its manufacturer, is not guaranteed or endorsed by the publisher.
